# Drug Delivery Implants in the Treatment of Vitreous Inflammation

**DOI:** 10.1155/2013/780634

**Published:** 2013-09-28

**Authors:** Jillian Wang, Angela Jiang, Malav Joshi, John Christoforidis

**Affiliations:** ^1^Department of Ophthalmology, The Ohio State University Wexner Medical Center, 915 Olentangy River Road, Suite 5000, Columbus, OH 43212, USA; ^2^Department of Ophthalmology & Visual Science, The University of Arizona Medical Center, 655 N. Alvernon Way, Suite 108, Tucson, AZ 85711, USA

## Abstract

The eye is a model organ for the local delivery of therapeutics. This proves beneficial when treating vitreous inflammation and other ophthalmic pathologies. The chronicity of certain diseases, however, limits the effectiveness of locally administered drugs. To maintain such treatments often requires frequent office visits and can result in increased risk of infection and toxicity to the patient. This paper focuses on the implantable devices and particulate drug delivery systems that are currently being implemented and investigated to overcome these challenges. Implants currently on the market or undergoing clinical trials include those made of nonbiodegradable polymers, containing ganciclovir, fluocinolone acetonide, triamcinolone acetonide, and ranibizumab, and biodegradable polymers, containing dexamethasone, triamcinolone acetonide, and ranibizumab. Investigational intravitreal implants and particulate drug delivery systems, such as nanoparticles, microparticles, and liposomes, are also explored in this review article.

## 1. Introduction

Posterior uveitis and vitreous inflammation can have devastating effects on vision. Treatment usually involves a long course of medication for adequate control of symptoms. Posterior subtenon or intravitreal injection of immunomodulators and steroids is short lasting, requiring frequent administration. Penetration of the posterior segment with topical and systemic agents can also prove challenging and can be associated with significant side effects [1]. With these limitations, efforts are being made to develop and implement implantable devices that slowly release drug into the vitreous. The anatomy of the eye makes it an excellent organ for such a drug delivery system.

Intravitreal drug delivery systems are coated with biodegradable or nonbiodegradable polymers. The main advantage of biodegradable implants is that they do not require removal. Properties of these polymers have been previously discussed in “Intravitreal devices for the treatment of vitreous inflammation [2].” This review describes current and investigational intravitreal drug delivery devices with a primary focus on their use in vitreous inflammation. Studies in other eye diseases such as macular edema, infection, and neovascularization are also included to illustrate the versatility of these devices and technologies and their potential application to vitreous inflammation.

## 2. Clinically Used Intravitreal Implants

### 2.1. Nonbiodegradable Devices

The nonbiodegradable devices are implanted into the vitreous surgically due to their large size, with the exception of Iluvien (Alimera Sciences Inc., Alpharetta, GA; pSivida Inc., Watertown, MA) which is smaller than the others. They usually require removal and reimplantation of a new device following depletion of the drug. These devices are made of a permeable membrane and include a drug-containing reservoir. Permeable and impermeable polymers can be layered to slow release of contents [3]. Thickness and surface area can also be manipulated to change the diffusion rate [4]. The first implantable device for clinical use in the eye was composed of a nonbiodegradable polymer containing ganciclovir. This drug delivery system, Vitrasert (Bausch & Lomb, Rochester, NY), was released to the market in 1996 for the treatment of acquired immunodeficiency syndrome- (AIDS-) related cytomegalovirus (CMV) retinitis [5, 6]. Also on the market is Retisert (Bausch & Lomb, Rochester, NY). Iluvien, I-vation (SurModics, Eden Prairie, MN), and the ranibizumab (Genentech, San Francisco, CA) port delivery system are still in the clinical trial phases for approval in the United States.

#### 2.1.1. Vitrasert

Vitrasert is a ganciclovir pellet coated in polyvinyl alcohol (PVA), a permeable polymer that allows drug diffusion, and ethylene vinyl acetate (EVA), an impermeable, a hydrophobic polymer that restricts release. It contains at least 4.5 mg of ganciclovir and 0.25% magnesium stearate as an inactive ingredient. The device is composed of outer and inner permeable PVA layers sandwiching a discontinuous layer of impermeable EVA. This combination of polymers results in a ganciclovir release rate of 1 mcg/hour that lasts 5 to 8 months before reimplantation is necessary [7].

Vitrasert has shown to be effective in treating CMV retinitis in AIDS patients, increasing median time to progression when compared to groups receiving intravenous ganciclovir [7]. More recent studies suggest that, in the era of highly active antiretroviral therapy, a ganciclovir implant device is less effective than systemic therapy at improving survival and decreasing dissemination [8].

Postoperative complications directly associated with Vitrasert implantation include cataract, vitreous hemorrhage, retinal detachment, endophthalmitis, and epiretinal membrane formation [9, 10].

#### 2.1.2. Retisert

Retisert is a sustained-release intravitreal implant containing 0.59 mg of fluocinolone acetonide coated with PVA and silicon laminate. Receiving FDA approval in 2005, it became the first intravitreal device for the treatment of chronic noninfectious uveitis. It is 5 mm long, 2 mm wide, and 1.5 mm thick with a release rate of 0.3-0.4 mcg/day for approximately 3 years. The device is inserted into the vitreous cavity and sutured to the sclera through a pars plana incision, a technique similar to the implantation of Vitrasert. Besides chronic noninfectious uveitis, studies have also shown the implant to be effective in edema from diabetes and central retinal vein occlusion (CRVO) [11, 12].

The clinical studies that resulted in the approval of Retisert found significant reduction of recurrence of uveitis, determined by anterior chamber cell number and vitreous haze, in patients treated for noninfectious posterior uveitis. In this three-year study, recurrence rate was significantly decreased from 62% in the year prior to implantation to 4%, 10%, and 20% in the 1st, 2nd, and 3rd year after implantation, respectively. Ocular complications encountered in implanted eyes, namely, lens opacification and increased intraocular pressure (IOP), were significant. Nearly 11% of implanted eyes required cataract extraction within the period of the study. In the same trial, 2-year safety data indicated that almost 100% of phakic patients would require cataract removal. This is a percentage much greater than that in patients with uveitis treated by steroids alone and indicates that the implant itself is contributing to increased lens opacity. By the end of the 3-year trial, 67% of implanted eyes had IOP elevated by 10 mmHg or more from baseline. Additionally, 49% required antihypertensive medication as compared to 13.6% at baseline. Other postoperative adverse events encountered included eye pain (52%), conjunctival hyperemia (31%), conjunctival hemorrhage (29%), hypotony (11%), retinal detachment (4%), and endophthalmitis (1%) [13, 14].

A recent randomized, controlled, phase 2b/3 trial demonstrated a lower rate of recurrence of uveitis in fluocinolone acetonide implanted eyes (18.2%) compared with those receiving standard of care, or systemic prednisolone or corticosteroid, treatment (63.5%). Observed adverse effects were similar to those in prior clinical trials. The systemically treated group, however, encountered nonocular adverse events (most commonly arthralgia and hypertension) of 25.7% compared to 0% in the implant group [15].

A large study enrolling 255 patients (479 eyes with uveitis), the multicenter uveitis steroid treatment (MUST) trial, also compared relative effectiveness of systemic therapy and fluocinolone acetonide implant in uveitis. It was shown that both approaches adequately controlled inflammation, but the implant group did so more often and earlier. Visual acuity was equally improved in both groups at the conclusion of the two-year study. As demonstrated in previous studies, eyes in the implant group had high complication rates with 80% requiring cataract surgery, 61% requiring treatment for IOP, and 16% with transient vitreous hemorrhage. Systemic treatment was well tolerated with no significant adverse events [16].

Another potential problem with the Retisert implant is the dissociation of the 2 main components (the suture strut and drug reservoir), which complicates removal and is potentially vision threatening. A retrospective study including 27 eyes found that 40.7% of the implants were dissociated at the time of removal or exchange [17].

#### 2.1.3. Iluvien

Iluvien is an intravitreal implant for the treatment of chronic diabetic macular edema (DME), defined as equal to or greater than 3 years of disease. It is 3.5 mm long by 0.37 mm wide and contains 190 mcg of fluocinolone acetonide. Due to its small size, it can be injected through a 25-gauge needle, creating a self-closing hole. The material is nonerodible and does not require removal, potentially resulting in multiple devices in the eye if subsequent implants are required. Besides use in DME, phase 2 studies in wet age-related macular degeneration (AMD), dry AMD, and retinal vein occlusion (RVO) are also in process. Iluvien is currently awaiting FDA approval following the recent completion of phase 3 clinical trials, also known as the fluocinolone acetonide for diabetic macular edema (FAME) trials.

The FAME study evaluated fluocinolone acetonide implants with release rates of 0.5 mcg/day and 0.2 mcg/day for 24–36 months. At 36 months, the percentage of patients who had gained at least 15 points in best-corrected visual acuity (BCVA) score was 28.7% in the low dose and 27.8% in the high dose implant groups compared to 16% in the sham group. Improvement of at least 2 lines in the early treatment of diabetic retinopathy study (ETDRS) acuity score was seen in a higher percentage of patients in the low dose group (13.7%) than in the sham group (8.9%). There was no significant difference in acuity between the high dose implant and sham. As with the Retisert implant, there was a high rate of increased IOP and cataract formation. Cataract surgery was performed in 80%, 87.2%, and 27.3% in the low dose, high dose, and sham groups, respectively. Adverse events related to IOP were more frequent in implant groups (low dose, 37.1%; high dose, 45.5%) compared to the shame (11.9%) [18]. These results indicate that the Iluvien implant, although with associated complications, is effective in DME, a disease that currently only has one FDA approved treatment.

#### 2.1.4. I-vation

I-vation is a helical sustained-release implant containing 0.925 mcg triamcinolone acetonide coated in titanium, PVA, and EVA. The implant elutes drug for up to 2 years. It measures 0.4 mm long by 0.21 mm wide and is implanted through a pars plana sclerotomy less than 0.5 mm in diameter. The helical shape is designed to increase surface area available for drug diffusion and anchor the device to the sclera, while the flat cap is meant to sit just beneath the conjunctiva. This facilitates removal of the implant if necessary.

Twenty-four-month interim results for phase 1 clinical trials of I-vation showed its effectiveness in treating DME. Macular thickness, measured by optical coherence tomography, was decreased and visual acuity improved. Major complications included increased IOP and cataract development [19]. Phase 2b trials were terminated, and no further clinical trials have been completed [20].

#### 2.1.5. Ranibizumab Port Delivery System

A novel port delivery system (PDS) with ranibizumab designed to release 10 mg/mL over an extended period of time is currently being investigated. A unique feature of this system is the ability to refill the device. A phase 1 uncontrolled clinical trial on neovascular age-related macular degeneration was recently completed in Latvia. The PDS, initially filled with 150 mcg of ranibizumab, resulted in improved visual acuity, sustained decrease in macular thickness, and evidence of decreased choroidal neovascular leakage comparable to monthly injections. Although final study data is pending, this technology shows promise in providing a long-term alternative to monthly ranibizumab injections [21].

### 2.2. Biodegradable Devices

Implants undergoing clinical trials in the United States for use in ocular disease include Surodex (Oculex Pharmaceuticals, Sunnyvale, CA) and Verisome (Icon Biosciences Inc., Sunnyvale, CA). A third, Ozurdex (Allergan Inc., Irvine, CA), is already approved for several indications. These devices are composed of biodegradable polymers that allow dissolution of the implant, eliminating the need for extraction and decreasing risks associated with surgery. There are currently two such devices on the market, both containing dexamethasone as the active ingredient.

#### 2.2.1. Ozurdex

Ozurdex is a dexamethasone-containing intravitreal implant coated in biodegradable poly(lactic-co-glycolic acid) (PLGA). The implant is a 6.5 mm by 0.45 mm rod placed in the vitreous through the pars plana with a 22-gauge needle device. It contains 0.7 mg of dexamethasone and releases peak doses for 2 months followed by a lower dose for up to 4 additional months. When compared to triamcinolone and fluocinolone, dexamethasone is 5 and 20 times more potent, respectively, but has a shorter half-life than either [22]. Ozurdex is approved for macular edema following BRVO or CRVO and noninfectious posterior uveitis.

In the HURON study, a 26-week multicenter, randomized clinical trial, 229 patients with noninfectious intermediate or posterior uveitis were randomized into groups receiving implants with 0.70 mg dexamethasone, 0.35 mg dexamethasone, or sham. Fifteen-letter improvement in BCVA was achieved in the dexamethasone groups at a rate 2- to 6-fold greater than that achieved in the sham. In addition to improvement in visual acuity, the mean decrease from baseline central macular thickness was also found to be greater in implant groups compared to sham at 8 weeks. They were not significantly different at 26 weeks. Percent of subjects with vitreous haze score of 0 at 8 weeks was 47%, 36%, and 12%, for those receiving high dose, low dose, and sham, respectively. This effect was maintained at 26 weeks. Adverse events included increased IOP with 23% of the 0.7 mg treatment group requiring medication to lower pressure and one patient requiring laser iridotomy. Cataract development was greater in treatment groups compared to the sham, but differences were not significant [23, 24]. When the device was implanted in patients with macular edema due to retinal vein occlusion in the GENEVA study, similar improvements in visual acuity were found. Unlike in the HURON study, occurrence of elevated IOP was not found to be significantly different between implanted and sham eyes by day 180 [25, 26].

The SOLO study, a retrospective chart study designed to compare results from the clinical setting to those of the GENEVA study, also showed improvement in visual acuity and reduction of macular edema. Early retreatment, defined as reinjection within the labeled 6-month interval, was performed in 40.7% and 50% of CRVO and BRVO eyes, respectively [27].

Several studies comparing the Ozurdex implant with intravitreal ranibizumab in retinal vein occlusion are ongoing [28–30]. Additionally, favorable outcomes have been demonstrated in small case series in patients with persistent uveitic cystoid macular edema with history of pars plana vitrectomy, radiation macular edema, and macular edema from retinitis pigmentosa [31–33].

#### 2.2.2. Surodex

Surodex is a 60 mcg dexamethasone pellet coated in PLGA and hydroxypropyl methylcellulose. It measures 1.0 mm by 0.4 mm and provides sustained release for 7–10 days following insertion into the anterior chamber. Surodex has completed phase 3 clinical trials in the United States and has been approved in China, Singapore, and several other countries. It has primarily been investigated as a treatment for postcataract surgery inflammation. One study showed that, over 7 days, the insert achieved higher concentration in the eye than the maximum peak concentrations reached with topical dexamethasone drops following cataract extraction [34]. A randomized clinical trial of Surodex as a steroid drug delivery system for cataract surgery showed the implant to be safe and effective in reducing postoperative inflammation. Their study included a group of subjects receiving two pellets in the anterior chamber, two pellets in the ciliary sulcus, and a control group that only received conventional topical 0.1% dexamethasone. Lower flare scores were experienced in both implant groups compared to the control without any difference between the two placements. No complications were encountered [35]. In a more recent study, Surodex was shown to be just as effective as topical 0.1% dexamethasone postcataract surgery without any significant improvement in decreasing flare. This study also reported no adverse events [36].

#### 2.2.3. Verisome

Verisome is an injectable drug delivery system that provides long-lasting intravitreal therapy. Once injected with a 30-gauge needle, the material coalesces to form a spherule that sits in the posterior chamber and slowly degrades as medication is released. According to the manufacturer, its versatility allows it to deliver small molecules, peptides, proteins, and monoclonal antibodies. It can, furthermore, be formulated as a gel, liquid, or solid [37].

A phase 1 multicenter study showed triamcinolone acetonide formulated with Verisome to be well tolerated and without injection-related complications such as endophthalmitis or uveitis. It was also demonstrated to be effective in improving chronic cystoid macular edema (CME) due to retinal vein occlusion [38]. A phase 2 clinical study for neovascular AMD with ranibizumab formulated with Verisome has also demonstrated its efficacy. Results of the study indicated that frequency of ranibizumab injections might be decreased with this drug delivery system [39].

## 3. Investigational Implants

### 3.1. Cyclosporine

Several animal studies have shown that intravitreal delivery of cyclosporine can help control inflammation of the posterior chamber. A PLGA cyclosporine microsphere delivery system significantly decreased severity of cellular infiltrate, leukocyte number, and protein levels in eyes of rabbits with uveitis without long-term toxicity [40]. A separate rabbit study demonstrated that cyclosporine A conjugated to a polycaprolactone (PCL)/PLGA copolymer was more effective in treating chronic uveitis when compared to oral cyclosporine [41]. Cyclosporine A contained in a 6 mm diameter suprachoroidal implant placed in the deep sclera has also been found to be effective in controlling inflammation and maintaining vision in an equine recurrent uveitis model [42].

### 3.2. Indomethacin

PLGA implants containing 7 mg of indomethacin released over 3 weeks were evaluated in a postoperative model in rabbits. Inflammation following capsulorhexis, phacoemulsification, and intraocular lens placement was significantly decreased, although the rate of posterior capsule opacification was unchanged from the control [43]. More recent studies focus on surface indomethacin implants in treating inflammation in the anterior chamber [44, 45].

### 3.3. Particulate Drug Delivery

Particulate drug delivery systems utilize small biodegradable colloidal particles for long-term delivery of medication. These systems also provide targeted therapy with improved bioavailability and decreased systemic toxicity. They include liposomes, microparticles, and nanoparticles. Microparticles and nanoparticles can further be subdivided into micro- or nanospheres, in which the drug is homogenously dispersed within a polymeric matrix, and micro- or nanocapsules, in which the drug is encased in a polymeric membrane. Distinction is based on particulate size with microparticles generally accepted as 1 to 1000 microns in diameter and nanoparticles between 10 and 1,000 nanometers [46].

#### 3.3.1. Liposomes

Liposomes are colloidal spheres made up of phospholipids, such as lecithin and phosphatidylcholine, which encapsulate therapeutic agents. Hydrophilic drugs are tucked away within the lipid core of the sphere, whereas hydrophobic drugs remain soluble between the bilayer. Because the phospholipids that compose these bilayers are naturally occurring, they are biocompatible with little toxicity and are capable of crossing hydrophobic membranes. Limitations of this delivery method include short half-life, instability, and minimal control of drug release over time [47]. Size of liposomes can also be engineered based on application. Those injected intravitreally are typically 100 nm to 400 nm in diameter according to the literature [48].

Use of liposomes as ocular drug delivery systems was first evaluated in superficial disease through topical instillation [49]. In more recent years, intravitreal administration has been under investigation. Currently, verteporfin (Visudyne, QLT Inc., Vancouver, BC, Canada), a benzoporphyrin derivative, is the only ophthalmic liposomal therapeutic agent approved. It is indicated in the treatment of neovascularization due to AMD, pathologic myopia, or presumed ocular histoplasmosis [50, 51]. Liposomal amphotericin or AmBisome (Gilead Sciences, Foster City, CA) is indicated for the treatment of leishmaniasis and various fungal infections in immunocompromised individuals and patients with renal impairment. It is also used off label for fungal endophthalmitis. The liposomal formulation has fewer toxic effects than the native form thus allowing delivery of higher dosages intravitreally [52]. Improvement of drug pharmacokinetic properties by intravitreal injection of liposomal therapeutics has also been observed in amikacin [53], amphotericin B [54], bevacizumab [55], cidofovir [56], ganciclovir [57], ciprofloxacin [58], clindamycin [59], gentamicin [60], and tobramycin [61].

#### 3.3.2. Microparticles

Microparticles are similar to liposomes in shape and size but have greater stability and capacity for carrying the drug. They are often composed of biodegradable polymers such as PLGA and polylactic acid (PLA). Surface polymer modification can also enhance specific cell targeting and decrease degradation by the mononuclear phagocytic system. The microparticles themselves, however, are not without risk. Unlike liposomes, the components of microparticles are not naturally found in the body and their exact interactions with living cells and tissue are not clearly understood [62]. To date, there are no microparticle drug delivery systems on the market. There are, however, many microparticle and nanoparticle therapeutic agents under investigation for improvement in long-term drug delivery.

Microcapsules containing TG-0054, a water soluble antiangiogenic drug in phase 2 clinical trials, were shown to sustain *in vivo* release for 3–6 months when injected in the vitreous. These PLA microparticles are, thus, a potentially useful formulation for long-term treatment of neovascular disorders of the eye [63].

Microspheres composed of PLGA and triamcinolone acetonide have shown potential utility in the treatment of DME in a nine-patient preliminary study. One mg triamcinolone acetonide in a controlled-release microsphere system (called RETAAC in the study) was well tolerated and demonstrated superior long-term pharmacological performance compared to a 4 mg injection of triamcinolone acetonide. No drug- or procedure-related side effects were noted in either group [64]. Other microspheres developed for sustained ocular delivery of agents include those containing adriamycin, pegaptanib, and cyclosporine [65–67]. Microspheres composed of chitosan, a natural biodegradable polymer, for transcorneal delivery of acyclovir have also demonstrated prolonged drug release [68].

#### 3.3.3. Nanoparticles

Nanoparticles have been used, experimentally, with several therapeutic agents for intraocular drug delivery. Injection of tamoxifen incorporated into polyethylene glycol- (PEG-) coated nanoparticles was found to be effective in the treatment of autoimmune uveoretinitis induced experimentally in rats. Injection of free tamoxifen, however, did not alter the course of disease [69]. Triamcinolone acetonide formulated in PLGA nanoparticles was recently studied in a rabbit model of endotoxin-induced uveitis. No significant difference existed between the effectiveness of triamcinolone acetonide injection and nanoparticles. Sustained-release nanoparticles, however, could potentially require fewer administrations and better patient compliance [70]. Intravitreally injected polyethylcyanoacrylate nanoparticles containing acyclovir and ganciclovir together showed sustained levels in rabbits but were also associated with cataracts and flare [71].

Nanoparticle technology has also been implemented in experimental gene transfer therapy. Periocularly injected recombinant pigment epithelium-derived factor (PEDF) particles significantly reduced choroidal neovascularization in mouse and pig models by increasing retinal PEDF levels [72, 73]. A similar technology was used in a phase 1 clinical trial of adenoviral vector-delivered PEDF in neovascular AMD with promising results. No serious adverse events were noted, but mild, transient intraocular inflammation did occur in 25% of patients and increased IOP in 21% [74]. Bevacizumab nanospheres composed of PLGA also demonstrated long-term release (over 90 days). Rate of release was adjusted by changing the drug to polymer ratio [75]. Topical cyclodextrin nanoparticles for the treatment of DME are currently being studied in a phase 2 clinical study in comparison to dexamethasone [76].

## 4. Summary

The structure of the eye makes it an organ that is well suited for local delivery of therapeutic agents. Several intravitreal devices are approved for inflammatory processes as well as other pathologic conditions of the eye. Intraocular nanoparticles and microparticles are also being developed and show great promise in sustained and targeted delivery of therapeutics. A multidisciplinary approach involving biomedical engineering, pharmacology, and molecular biology will continue to be critical in the design of implants for the treatment of ocular inflammation. Many of the discussed drug delivery devices have varying benefits and limitations. As knowledge of these delivery systems and implants broadens, a safe and efficacious device that does not necessitate removal or surgical implantation may, in the future, be available as standard treatment for many ocular diseases.
